# Transcriptome analysis reveals molecular mechanisms underlying salt tolerance in halophyte *Sesuvium portulacastrum*

**DOI:** 10.3389/fpls.2022.973419

**Published:** 2022-09-23

**Authors:** Dan Wang, Nan Yang, Chaoyue Zhang, Weihong He, Guiping Ye, Jianjun Chen, Xiangying Wei

**Affiliations:** ^1^Institute of Oceanography, College of Geography and Oceanography, Minjiang University, Fuzhou, China; ^2^Fuzhou Institute of Oceanography, Fuzhou, China; ^3^Department of Environmental Horticulture, Mid-Florida Research and Education Center, Institute of Food and Agricultural Sciences, University of Florida, Apopka, FL, United States

**Keywords:** salt tolerance, *Sesuvium portulacastrum*, transcriptomic analysis, differentially expressed genes, ion transport

## Abstract

Soil salinity is an important environmental problem that seriously affects plant growth and crop productivity. Phytoremediation is a cost-effective solution for reducing soil salinity and potentially converting the soils for crop production. *Sesuvium portulacastrum* is a typical halophyte which can grow at high salt concentrations. In order to explore the salt tolerance mechanism of *S. portulacastrum*, rooted cuttings were grown in a hydroponic culture containing ½ Hoagland solution with or without addition of 400 mM Na for 21 days. Root and leaf samples were taken 1 h and 21 days after Na treatment, and RNA-Seq was used to analyze transcript differences in roots and leaves of the Na-treated and control plants. A large number of differentially expressed genes (DEGs) were identified in the roots and leaves of plants grown under salt stress. Several key pathways related to salt tolerance were identified through KEGG analysis. Combined with physiological data and expression analysis, it appeared that cyclic nucleotide gated channels (CNGCs) were implicated in Na uptake and Na^+^/H^+^ exchangers (NHXs) were responsible for the extrusion and sequestration of Na, which facilitated a balance between Na^+^ and K^+^ in *S. portulacastrum* under salt stress. Soluble sugar and proline were identified as important osmoprotectant in salt-stressed *S. portulacastrum* plants. Glutathione metabolism played an important role in scavenging reactive oxygen species. Results from this study show that *S. portulacastrum* as a halophytic species possesses a suite of mechanisms for accumulating and tolerating a high level of Na; thus, it could be a valuable plant species used for phytoremediation of saline soils.

## Introduction

Soil salinization is a soil degradation process and considered as one of the most important global issues (Zelm et al., 2020). Excessive salt, mainly sodium (Na) can affect the decomposition of soil aggregates, deplete soil nutrients, and lead to deteriorate soil structure and soil quality, thereby threatening crop production and the environment (Qadir et al., 2001). Globally, about 831 million hectares of land were affected by salinization (Butcher et al., 2016), of which 23.35% is located in Asia (FAO, 2015). Currently, soil salinization takes up to 1.5 million hectares of farmland per year out of crop production (FAO, 2015). Land restoration under the influence of severe salinization is the key to land resource management and sustainable development. Among the strategies, phytoremediation is regarded as an important strategy for the restoration of saline soils because of its cost-effective and environmentally friendly characteristics (Ashraf et al., 2010; Imadi et al., 2016). Halophytes are naturally salt-tolerant plants and have been used in the reclamation of saline-alkali land based on the characteristics of salt accumulation in their leaves and stems (Ayyappan and Ravindran, 2014; Hasanuzzaman et al., 2014; Hayat et al., 2020). Exploring salt tolerance mechanisms of halophytes could assist in our effort on the use of this group of plants for remediation of saline soils.

Halophytes usually live in intertidal zone or inland saline soil. Unlike glycophytes, halophytes can complete their life cycle when grown in seawater or high-salt soils (Chen et al., 2016). Salt stress generally causes excessive accumulation of Na^+^ in plants, resulting in osmotic stress and ion toxicity and inhibiting plant growth (Hasegawa et al., 2000; Rana and Mark, 2008). To adapt to the high-salt environments, while sharing the salt tolerance mechanism with glycophytes, halophytes have evolved special features. For example, osmotic protection is a general response to salt that maintains cell osmotic pressure and turgor pressure through osmotic regulation or ion membrane transport (Singh et al., 2015). Primary and secondary metabolites, including proline and soluble sugars, play an osmotic adjustment function under salt stress (Chen and Jiang, 2010). On the other hand, to deal with ionic toxicity and maintain Na^+^/K^+^ homeostasis in the cell, plants develop mechanisms by limiting the excessive accumulation of Na^+^ in the cytoplasm, including the restriction of the entry of Na^+^ into cells, removal of Na^+^ from the cells, and storing excess Na^+^ into the vacuole (Zhao et al., 2020). Some important ion transporters include SOS1, HKT1, and NHXs, are involved in the removal of Na^+^ from the cytoplasm, the transport of Na^+^ from root cells to xylem, and ion isolation in vacuoles (Shi et al., 2000; Davenport et al., 2010; Apse et al., 2019). Compared with glycophytes, most halophytes can accumulate more Na^+^ in the aerial portion under salt stress and can maintain a better Na^+^/K^+^ ratio (Orsini et al., 2010; Flowers et al., 2015).

*Sesuvium portulacastrum*, as a mangrove companion plant, is a typical halophyte in the family *Aizoaceae* and usually grows on wet sand, such as beaches (Lonard and Judd, 1997; He et al., 2022; Zhang et al., 2022). *Sesuvium portulacastrum* plants are tolerant to salt, drought, and heavy metal stresses and widely used in phytoremediation projects including salt-alkali soil restoration in coastal areas and coastal sand fixation (Lokhande et al., 2013). Early studies have shown that *S. portulacastrum* is a facultative halophyte and salt accumulator, suggesting that *S. portulacastrum* plants can grow well in not only non-saline but also saline soils. Appropriate saline levels could even increase the photosynthetic rate of *S. portulacastrum* plants and promote their growth (Peng et al., 2018). A high salt concentration (200 mM NaCl) significantly alleviated Cd toxicity symptoms in *S. portulacastrum* by both limiting Cd uptake and compartmenting Cd inside plant tissues (Mariem et al., 2014)*. Sesuvium portulacastrum* plants are able to sustain their growth by isolating salt ions into the vacuole to maintain the osmotic balance between the vacuole and the cytoplasm (Ramani et al., 2006). A quantitative proteomic analysis identified 96 salt-responsive proteins implicated in salt stress in *S. portulacastrum*, and these proteins are highly involved in ion binding, proton transport, photosynthesis, and ATP synthesis. Under high salinity conditions, the expression of Na^+^/H^+^ antiporter and ATP synthase subunits was activated (Yi et al., 2014). Enzymatic analyses showed that NaCl could induce *S. portulacastrum* P-ATPase and V-ATPase activities, but V-PPase activity was inhibited (Ding et al., 2022). Several genes in *S. portulacastrum* have been demonstrated to promote salt tolerance in plants. For example, an aquaporin gene *SpAQP1* from *S. portulacastrum*, increased salt tolerance in transgenic tobacco (Chang et al., 2016). Heterologous overexpression of *SpSOS1* and *SpAHA1* genes improved salt tolerance in yeast and *Arabidopsis* (Zhou et al., 2015, 2018). With the increasing recognition of *S. portulacastrum* as an important species for phytoremediation of saline soils and water body, there is a need to further study of this species in tolerance to saline environments. Understanding its tolerance to salinity at the molecular level would enable us to better utilize this and other halophytic plants for remediation of saline soils.

The objectives of this study were to (1) evaluate morphological responses of *S. portulacastrum* plants to different concentrations of Na in a hydroponic culture, (2) analyze transcript changes of the plants after exposure to a high level of Na in contrast with those without Na treatment through RNA-Seq, (3) identify important genes involved in the tolerance of high Na concentration, (4) outline the major strategies of the plants in adaptation to saline conditions and maintenance of plant growth. It was anticipated that this effort could provide a global view of the transcript changes in and major routes for *S. portulacastrum* to adapt to saline environments, and such information could assist in our effort on improving crop tolerance of salt stress.

## Materials and methods

### Plant growth and salt treatments

The shoots of *S. portulacastrum* were initially collected from Putian, a coastal city in Fujian Province, China. They were potted in containers filled with a substrate composed of 40% peat, 20% pine bark, 20% perlite, and 20% sand based on volume (Zhonghe Agriculture, Huaian, China) and grown in a greenhouse at the Minjiang University, Fuzhou, Fujian, China. Tip cuttings with four nodes and about 10 leaves were made. After removing leaves from the lowest node, they were rooted in ½ Hoagland solution (Hoagland and Synder, 1933). Uniform rooted cuttings were selected, and four cuttings were planted in each 3-L container filled with 2 L ½ Hoagland solution supplemented with NaCl resulting in Na concentrations at 0, 100, 200, 300, 400, 500, and 600 mM, respectively. The plants were grown in a growth room with a constant temperature of 24°C under a light intensity about 400 μmol/m^2^/s and a photoperiod of 16 h. The experiment was arranged as a completely randomized design with three replications. Plant growth was monitored every 3 days for 21 days by taking each plant out of the solution culture and carefully blotting with paper towel, and fresh weight and root numbers were recorded (Supplementary Table 1). Plant growth photos were taken on days 0, 12, and 21, and growth curves based on root numbers and fresh weight produced under different concentrations of Na were drawn at the end of experiment.

### RNA extraction and sequencing

Based on the results of above experiment, 400 mM Na was selected for the second solution culture experiment to determine transcript changes in *S. portulacastrum* plants. Rooted cuttings were grown in the same containers filled with ½ Hoagland solution devoid of Na or containing 400 mM Na. The experiment was set as a completely randomized experiment with three replications. Root and leaf samples were taken 1 h and 21 days after the initiation of the experiment. The samples were taken from three containers per treatment, respectively, and frozen immediately in liquid nitrogen. Thus, there were three biological replications for each treatment. After 21 days, entire plants were removed from each container, washed with deionized water three times, blotted with paper towel. Leaves and roots were separated, placed in paper bags, and dried at 105°C for 2 h, and then dried at 80°C for 48 h. Dry weight of each sample was weighed, which were used for analysis of Na^+^ and K^+^ contents.

A portion of the frozen leaf and root samples were sent to Novogene Co. (Beijing, China) for RNA extraction and sequencing. RNA was isolated from roots and leaves, respectively, and they were sequenced separately. There were three biological replicates for each sample. The RNA integrity and total concentrations were analyzed using Agilent 2100 BioAnalyzer (Agilent Technologies, Palo Alto, CA, United States). 1 μg of RNA from each sample was taken for library construction. Agilent 2100 BioAnalyzer was used to determine the insert size of the library. The qRT-PCR was used to accurately quantify the library effective concentration to ensure the quality of the library. The prepared libraries were sequenced by the Illumina HiSeq 2000 sequencing system (Novogene Co., Beijing, China) to generate 150 bp paired end readings. To obtain the clean data, reads with adapter, reads containing N and low-quality reads (reads with a Qphred <=20 base number) were removed from the raw data. The sequence data was submitted to the National Center for Biotechnology Information (NCBI) with an accession number of PRJNA848266.

### Differential expression analysis

Trinity (v2.5.1; Grabherr et al., 2011) was used for transcriptome assembly. Clean reads were mapped on the transcriptome to obtain the read count of each gene using the RSEM software (Dewey and Li, 2011). The read count of genes was converted to FPKM (fragments per kilobase of transcript per million fragments mapped). Differential expression analysis of the two treatments (0.0 mM vs. 400.0 mM at each sampling time) was performed using DESeq2 R package (*v1.20.0*) based on padj <0.05 and | log2FoldChange | > 1 (Love et al., 2014). KEGG pathway enrichment analysis of the differential expression genes was conducted using KOBAS software (version 3.0; http://kobas.cbi.pku.edu.cn/). Gene functional annotation was performed using the following five databases: Non-Redundant Protein Sequence Database (Nr), Nucleotide Sequence Database (Nt), Protein family (Pfam), Clusters of Orthologous Groups of proteins (KOG/COG), Swiss-Prot, Kyoto Encyclopedia of Genes and Genomes (KEGG), and Gene Ontology (GO). Furthermore, the top 5,000 differentially expressed genes of FPKM were selected for WGCNA analysis. The WGCNA R package was used for co-expression network analysis (Langfelder and Horvath, 2009).

### qRT-PCR analysis

To verify the expression of identified genes, RNA was extracted from roots and leaves of *S. portulacastrum* grown in 0 or 400 mM Na (A portion of the remaining frozen samples mentioned above) using an Omega plant RNA kit (Omega, Bio-Tek Inc. Norcross, GA, United States). After analysis of the quality of RNA, the first strand of cDNA was synthesized with a FastKing RT Kit (Tiangen, Beijing, China) using 1 μg RNA. For qRT-PCR analysis, the cDNA was diluted fivefold with water. The reaction solution for qRT-PCR included 0.8 μl forward primer and 0.8 μl reverse primer, 1 μl cDNA, 10 μl of 2 × SYBR Premix Ex Taq (Takara, Japan) and 7.4 μl deionized water. The reaction program was composed of 95°C for 3 min, then 95°C for 10 s, 60°C for 15 s, and 72°C for 20 s with a total of 45 cycles. qRT-PCR was carried out on an iCycler iQ5 thermal cycler (Bio-Rad, Hercules, CA, United States). The *S. portulacastrum SpGAPDH* was used as internal control (Zhou et al., 2015). Three biological replicates were performed for each sample. Gene relative expression levels were calculated using the 2^−∆∆Ct^ method (Livak and Schmittgen, 2002). Primers used for the study are listed in Supplementary Table 2.

### Analysis of reduced glutathione, soluble sugar, proline, K, and Na

The contents of reduced glutathione (GSH), soluble sugar, and proline in leaves and roots of *S. portulacastrum* were measured according to the instructions of the commercial kits (Nanjing Jiancheng Bioengineering Institute, Nanjing, China). Fresh plant samples were ground with liquid nitrogen. Precisely weighted each sample was added to PBS buffer with a ratio of 1:9 (g:ml) and vortexed. The solution was centrifuged for 10 min (2,500 rpm), and resultant supernatant was used for testing. Reagents were added according to the manufacturer instructions. The contents of GSH, soluble sugar, and proline were analyzed by a microplate reader with the absorbance at 405, 620, and 520 nm, respectively.

For analysis of Na^+^ and K^+^, the aforementioned dried leaf and root samples harvested at the end of the experiment were digested using the method described by Haynes (1980). The inductively coupled plasma (ICP)-atomic emission spectrometry (Perkin Elmer Instruments, Shelton, CT, United States) was used to measure Na^+^ and K^+^ content (Plank, 1992). Three biological replicates were analyzed per treatment.

## Results

### Plant growth responses to different concentrations of Na

**Figure 1:**
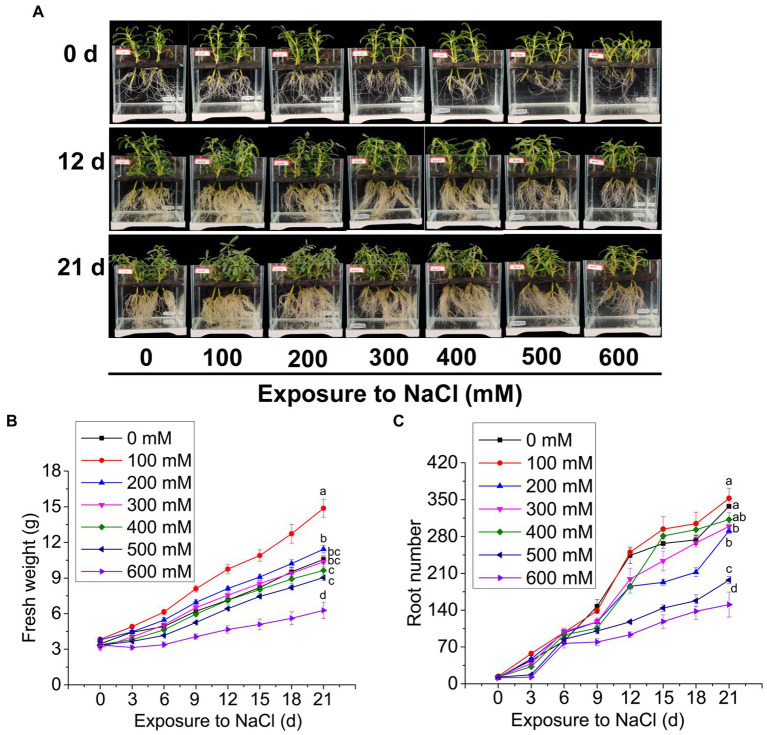
The growth of *Sesuvium portulacastrum* in a hydroponic culture. The phenotype of *S. portulacastrum* plants grown in different concentrations of NaCl over 0, 12, and 21 days **(A)**, fresh weight **(B)**, and root numbers **(C)** of plants over 21 days of growth. Data were means of three replications. Different letters at the end of lines indicate significant differences among treatments based on Tukey HSD test at *p* < 0.05 level.

Rooted cuttings of *S. portulacastrum* were able to sustain their growth over a 21-day period regardless of Na concentrations (Figure 1A). Fresh weights of plants exposed to Na at a concentration ranging from 100 to 500 mM were either comparable to or higher than those of the control plants (Figure 1B). The highest fresh weights were produced when plants were exposed to 100 mM Na. However, when exposed to 600 mM Na, the magnitude of fresh weight increase over the time was significantly lower than the other treatments, indicating growth suppression. Root numbers of plants grown at 0 and 100 mM Na were the highest, followed by plants grown with Na at 200–400 mM (Figure 1C). Root numbers of plants grown at 500 mM Na were significantly lower than those grown in the other treatments except for plants grown at 600 mM that had the lowest number of roots.

### Global transcriptome analysis of *S. portulacastrum* under salt stress

**Figure 2:**
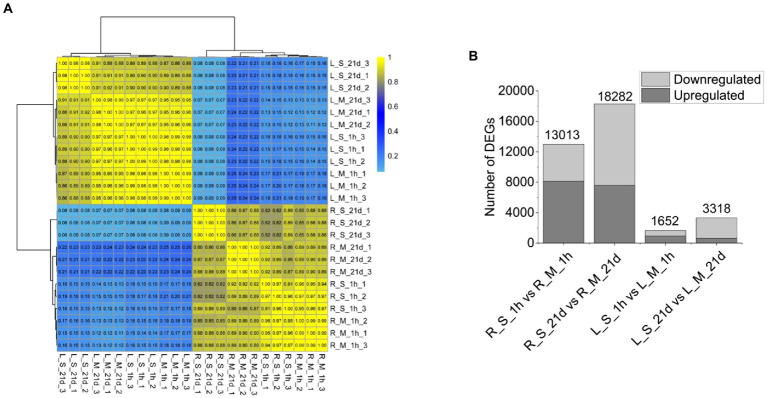
RNA-Seq data and DEGs resulted from *S. portulacastrum* plants treated and untreated with 400 mM NaCl. **(A)** Hierarchical clustering of 24 samples based on the correlation coefficient (*r^2^*), where R_M_1h and R_M_21d were samples of roots (1 h and 21 days) without NaCl treatment; L_M_1h and L_M_21d were samples of leaves (1 h and 21 days) without NaCl treatment; R_S_1h and R_S_21d were samples of roots (1 h and 21 days) with NaCl treatment; and L_S_1h and L_S_21d were samples of leaves (1 h and 21 days) with NaCl treatment. **(B)** Statistics of DEGs numbers in roots and leaves.

Since the fresh weights and root numbers were similar between plants grown at 0 mM and 400 mM Na (Figure 1), leaf and root samples of plants grown at the two levels of Na were analyzed using RNA-Seq. We obtained 152.28 Gb of filtered sequence data, of which 90.76% of the data quality reached Q30 or higher (Supplementary Table 3). Because the genome of *S. portulacastrum* has not been released yet, the reads were mapped to the transcriptome assembled with Trinity (*v2.5.1*). The BUSCO software was used to evaluate the quality of “cluster.fasta” file obtained by assembling. The comparison rate of complete transcripts (single copy and duplicated copies) reached 55.2% (Supplementary Table 4). All the genes were clustered according to the correlation coefficient (*r^2^*). Genes from roots and leaves were separately clustered, and three biological replicates of the same treatment were clustered together (Figure 2A). The results showed that replicate samples were highly correlated. In order to identify differentially expressed salt-responsive genes in roots and leaves, respectively, we compared the expression levels between the following pairs: R_S_1h vs. R_M_1h (R_S_1h represented roots treated with Na for 1 h, and R_M_1h was roots without exposure to Na for 1 h); R_S_21d vs. R_M_21d (R_S_21d, roots treated with Na for 21 days, and R_M_21d, roots without Na treatment for 21 days); L_S_1h vs. L_M_1h (L_S_1h, leaves of plants treated with Na for 1 h, and L_M_1h, leaves of plants without Na treatment); and L_S_21d vs. L_M_21d (L_S_21d, leaves of plants treated with Na for 21 days, and L_M_21d, leaves of plants without Na treatment for 21 days). Compared with the control, a total of 13,013 differentially expressed genes (DEGs) were identified in the roots, of which 8,135 were up-regulated and 4,878 were down-regulated. Whereas 1652 DEGs were identified in leaves, of which 929 were up-regulated, and 723 were down-regulated after 1 h of salt treatment. For plants exposed to 400 mM for 21 days, 18,282 DEGs were identified in the roots (7,584 up-regulated and 10,698 down-regulated) and 3,318 DEGs were identified in leaves (624 were up-regulated, and 2,694 were down-regulated; Figure 2B). These results indicated that the expression of salt-responsive genes varied significantly between roots and leaves, and the expression levels were affected by the duration of the treatments.

### Stress-responsive genes of *S. portulacastrum* enriched under salt stress

**Figure 3:**
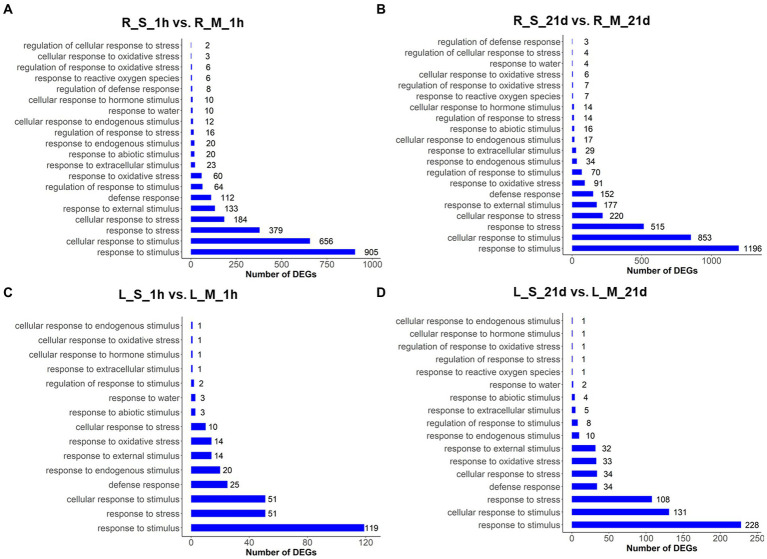
GO analysis of DEGs in roots and leaves of plants treated and untreated with 400 mM Na. The GO terms were identified based on DEGs of different comparisons: R_S_1h vs. R_M_1h **(A)**; R_S_21d vs. R_M_21d **(B)**; L_S_1h vs. L_M_1h **(C)**; L_S_21d vs. L_M_21d **(D)**. The bars represent the number of DEGs in each GO term.

To further explore the changes in gene expression in *S. portulacastrum* under salt stress, GO enrichment analysis was performed with DEGs by pair comparisons: R_S_1h vs. R_M_1h; R_S_21d vs. R_M_21d; L_S_1h vs. L_M_1h; and L_S_21d vs. L_M_21d (Figures 3A–D). Several GO terms related to stress responses were identified in both roots and leaves, such as response to stimulus, response to stress, and response to oxidative stress. But some GO terms were not identified in leaves after salt treatment for 1-h and 21-day, such as response to reactive oxygen species and cellular response to oxidative stress (Figure 3). More DEGs were identified in roots (Figures 3A,B) than leaves (Figures 3C,D), indicating the numbers of stress-responsive genes in roots were significantly higher than those in leaves.

### Genes related to ion transport in *S. portulacastrum*

A large number of DEGs related to ion transporters were identified (Table 1), which included four Na^+^/K^+^ transporters (*HKTs*), 10 shaker-type K^+^ channels (*AKT1s*), two high affinity potassium (*HAKs*), 12 cyclic nucleotide gated channels (*CNGCs*), 47 aquaporins, and nine glutamate receptors (*GLRs*). At the same time, multiple DEGs involved in Na^+^ extrusion and sequestration were identified, including four Na^+^/H^+^ exchangers (*NHXs*) and 71 H^+^-ATPases. In addition, multiple cation channel genes were detected, including five K^+^ efflux antiporters (*KEAs*), three K^+^ uptake permeases (*KUPs*), two pore potassium channels (*TPKs*), two Ca^2+^/H^+^ antiporters, and nine cation/H^+^ antiporters (Supplementary Table 5). In general, the expression of DEGs involved in Na^+^ extrusion and sequestration increased after being exposed to Na stress.

**Table 1:** Differentially expressed ion transporters in roots and leaves under salt stress.

Cluster ID	Database description	log_2_ (Fold change)			
		Root_1h	Root_21d	Leaf_1h	Leaf_21d
Na^+^ uptake					
Cluster-51977.13165	Glutamate receptor (GLR)	−1.5703	1.3975	–	–
Cluster-51977.5676	Glutamate receptor (GLR)	−3.1902	−9.2789	–	–
Cluster-51977.32649	Glutamate receptor (GLR)	–	1.7076	–	–
Cluster-51977.87644	Glutamate receptor (GLR)	1.3652	–	–	–
Cluster-51977.137910	Glutamate receptor (GLR)	–	1.8601	–	–
Cluster-51977.81862	Cyclic nucleotide-gated ion channel (CNGC)	1.0804	–	–	1.0344
Cluster-51977.81878	Cyclic nucleotide-gated ion channel (CNGC)	1.9346	−1.1817	–	–
Cluster-51977.100764	Cyclic nucleotide-gated ion channel (CNGC)	–	−1.0047	–	–
Cluster-51977.51402	Cyclic nucleotide-gated ion channel (CNGC)	1.6637	–	–	–
Cluster-51977.51403	Cyclic nucleotide-gated ion channel (CNGC)	1.4292	–	–	–
Cluster-51977.131278	Na^+^/K^+^ transporter (HKT)	–	−4.1136	–	–
Cluster-51977.72258	Na^+^/K^+^ transporter (HKT)	–	−3.2744	–	–
Cluster-51977.70693	Na^+^/K^+^ transporter (HKT)	–	−3.1004	–	–
Cluster-51977.12531	Na^+^/K^+^ transporter (HKT)	–	−4.6243	–	–
Cluster-51977.102438	Shaker-type K^+^ channels AKT1	–	−1.6052	–	–
Cluster-51977.93455	Shaker-type K^+^ channels AKT1	−1.135	1.5776	–	–
Cluster-51977.102439	Shaker-type K^+^ channels AKT1	–	−1.7222	–	–
Cluster-51977.87709	Shaker-type K^+^ channels AKT1	−1.2702	1.5352	–	–
Cluster-51977.24379	Shaker-type K^+^ channels AKT1	–	–	–	−1.0309
Cluster-51977.68836	High affinity potassium (HAK)	–	1.306	–	–
Cluster-51977.83444	High affinity potassium (HAK)	–	1.3255	–	–
Cluster-51977.100439	Aquaporin TIP	−1.3463	–	–	–
Cluster-51977.44303	Aquaporin PIP	−1.2535	–	–	–
Cluster-51977.65599	Aquaporin PIP	–	–	1.0919	–
Cluster-51977.70411	Aquaporin TIP	−1.47	–	–	−1.1722
Cluster-51977.53002	Aquaporin PIP	–	−2.0825	–	–
Na^+^ extrusion and Na^+^ sequestration					
Cluster-51977.50507	Na^+^/H^+^ exchanger (NHX)	1.1936	–	1.2854	–
Cluster-51977.37461	Na^+^/H^+^ exchanger (NHX)	1.219	–	1.0589	–
Cluster-51977.50509	Na^+^/H^+^ exchanger (NHX)	2.4805	–	1.6241	–
Cluster-69520.0	Na^+^/H^+^ exchanger (NHX)	–	5.7103	–	–
Cluster-51977.72274	Plasma membrane H^+^-ATPase	–	1.1546	–	–
Cluster-51977.106274	Plasma membrane H^+^-ATPase	–	1.6041	–	–
Cluster-51977.72273	Plasma membrane H^+^-ATPase	–	1.4131	–	–
Cluster-51977.150164	Plasma membrane H^+^-ATPase	–	9.8178	–	–
Cluster-41429.0	Plasma membrane H^+^-ATPase	−2.9645	–	−2.9645	–
K^+^ channel					
Cluster-51977.78847	K^+^ efflux antiporter (KEA)	–	–	–	−1.2637
Cluster-51977.73195	K^+^ efflux antiporter (KEA)	–	−1.059	–	–
Cluster-51977.78854	K^+^ efflux antiporter (KEA)	–	–	–	−1.4937
Cluster-51977.128334	K^+^ efflux antiporter (KEA)	–	1.687	–	–
Cluster-36451.0	K^+^ efflux antiporter (KEA)	−3.8638	–	–	–
Cluster-51977.9496	K^+^ uptake permease (KUP)	−1.4907	−5.7325	–	–
Cluster-51977.5805	K^+^ uptake permease (KUP)	−4.4418	−2.8311	–	–
Cluster-51977.65788	K^+^ uptake permease (KUP)	–	1.4587	–	–
Cluster-51977.32311	Two pore potassium channel (TPK)	–	−1.5353	–	−1.0312
Cluster-51977.32312	Two pore potassium channel (TPK)	–	−2.003	–	–
Other cation channels					
Cluster-29290.0	Ca2^+^/H^+^ antiporter	−2.9047	–	–	–
Cluster-51977.27292	Ca2^+^/H^+^ antiporter	–	−1.0955	–	–
Cluster-51977.101674	Cation/H^+^ antiporter	1.0254	–	1.1723	–
Cluster-51977.61674	Cation/H^+^ antiporter	−2.0507	−3.4027	–	−4.1648
Cluster-51977.111068	Cation/H^+^ antiporter	–	2.6856	–	1.5372
Cluster-51977.24139	Cation/H^+^ antiporter	2.2506	–	–	–
Cluster-51977.109616	Cation/H^+^ antiporter	1.2815	2.4522	–	–

The data is from RNA-seq. “–” means the gene is not expressed.

**Figure 4:**
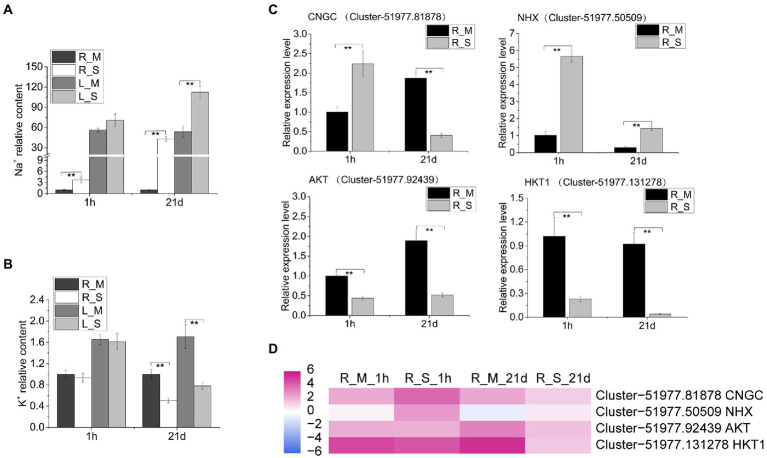
Tissue concentrations of Na and K and qRT-PCR analysis of relevant gene expression. **(A,B)** The relative content of Na and K in roots and leaves of plants after 1 h and 21 days of growth with 400 mM Na compared to the R_M sample. R_M and L_M mean roots and leaves of plants unexposed to Na. R_S and L_S mean roots and leaves of plants exposed to 400 mM Na. **(C)** qRT-PCR analysis of the expression of Na^+^ and K^+^ transport related genes. The error bars were calculated from three biological replicates. Mean differences were analyzed based on Tukey’s HSD test at *p* < 0.01 (^**^) levels. **(D)** The expression of genes (CNGC, NHX, AKT, and HKT1) in roots of plants treated with or without 400 mM Na. The data represent log_2_ FPKM values based on RNA-Seq analysis.

The analysis of Na^+^ and K^+^ contents in roots and leaves showed that the Na^+^ content increased significantly in roots and leaves after salt treatment. Na^+^ contents in roots and leaves reached 58.11 and 152.05 mg/g, respectively, after 21 days of salt treatment. Compared with the control samples at the same period, the Na content in the roots and leaves increased by 42.9 folds and 2.1 folds, respectively (Figure 4A). The K^+^ contents in *S. portulacastrum* decreased after 21 days of salt treatment, and the contents in roots and leaves were 17.60 and 27.37 mg/g, respectively. Compared with the control samples at the same period, the K content in the roots and leaves was reduced by 0.5 and 0.43 folds, respectively (Figure 4B). We further used qRT-PCR to analyze the expression levels of ion transport related genes in roots. The expression level of *CNGC* increased significantly after 1 h of salt stress, and decreased after 21-day. The expression level of NHX was significantly increased relative to the control after salt treatment for 1-h and 21-day (Figure 4C). In addition, the expression of K^+^ transport related genes *AKT* and *HTK1*, was inhibited under salt stress (Figure 4C). The quantitative expression trends of these genes were consistent with the trends in RNA-Seq (Figure 4D).

### Metabolic pathways related to salt stress in *S. portulacastrum*

**Figure 5:**
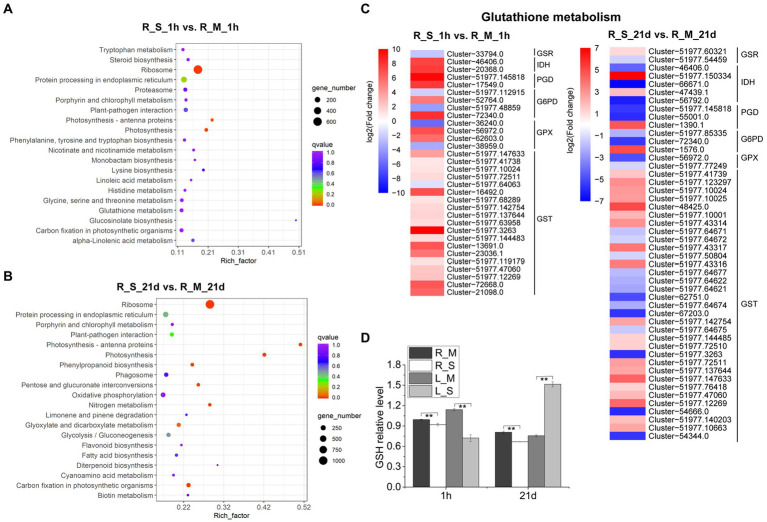
KEGG analysis of DEGs in roots and expression analysis of DEGs involved in “glutathione (GSH) metabolism.” **(A)** The top 20 pathways that were significantly enriched in roots after 1 h of salt treatment (400 mM Na). **(B)** The top 20 pathways that were significantly enriched in roots after 21 days of salt treatment. **(C)** Expression analysis of DEGs in roots involved in “Glutathione metabolism” after 1 h and 21 days of salt treatment. **(D)** The relative content of GSH in roots and leaves compared to the R_M sample. R_M and L_M mean roots and leaves of plants untreated with Na. R_S and L_S mean roots and leaves of plants treated with 400 mM Na. The error bars were calculated from three biological replicates. Mean separation was based on Tukey HSD test at *p* < 0.01 (^**^) levels.

In order to explore metabolic pathways involved in salt stress, a pathway enrich analysis was conducted using KEGG. We analyzed DEGs in roots of *S. portulacastrum* under salt stress and found that 20 pathways were significantly enriched, including photosynthesis – antenna proteins, alpha-linolenic acid metabolism, lysine biosynthesis, and GSH metabolism (Figures 5A,B). Among them, GSH metabolism was among the most enriched pathways. As a result, the expression levels of genes involved in GSH metabolism were analyzed. Results showed that after 1-h salt treatment, a large number of glutathione S-transferase (GST) genes were highly up-regulated in roots. In addition, genes involved in catalyzing the production of oxidized glutathione (GSSG) from GSH, such as isocitrate dehydrogenase (IDH), 6-phosphogluconate dehydrogenase (PGD), glucose-6-phosphate 1-dehydrogenase (G6PD), and glutathione peroxidase (GPX) were mostly up-regulated (Figure 5C). However, after 21-day salt treatment, the expression of these DEGs in roots were largely down-regulated compared to those after 1-h salt treatment (Figure 5C). We further analyzed the GSH content and found that salt treatment led to a reduction of the GSH content in the *S. portulacastrum* roots (Figure 5D).

**Figure 6:**
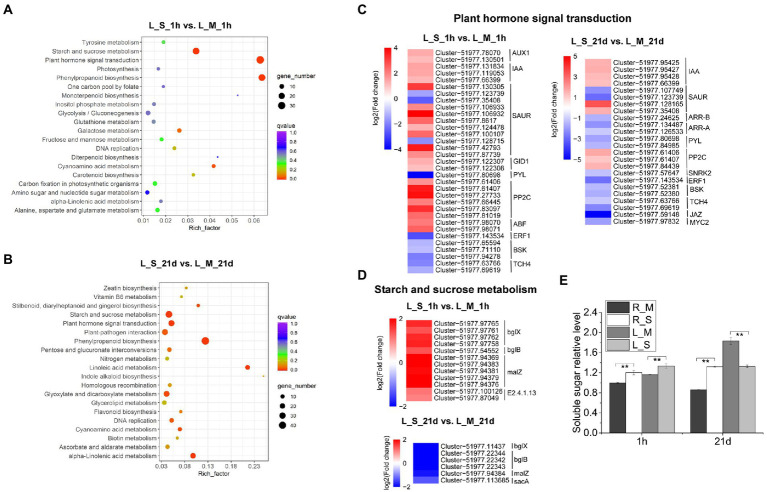
KEGG analysis of DEGs in leaves and expression analysis of DEGs involved in “plant hormone signal transduction” and “starch and sucrose metabolism.” **(A)** The top 20 pathways that were significantly enriched in leaves of plants after 1 h of salt treatment (400 mM Na). **(B)** The top 20 pathways that were significantly enriched in leaves after 21 days of salt treatment. **(C,D)** Expression analysis of DEGs in leaves involved in “plant hormone signal transduction” and “starch and sucrose metabolism” after 1 h and 21 days of salt treatment. **(E)** The relative content of soluble sugar content in roots and leaves compared to the R_M sample. R_M and L_M mean roots and leaves of plants untreated with Na. R_S and L_S mean roots and leaves of plants treated with 400 mM Na. The error bars were calculated from three biological replicates. Mean separation was based on Tukey HSD test at *p* < 0.01 (^**^) levels.

The KEGG pathway enrichment was also used for analysis of DEGs in leaves, and 20 significantly enriched pathways were identified, including plant hormone signal transduction, phenylpropanoid biosynthesis, starch and sucrose metabolism, and GSH metabolism (Figures 6A,B). Considering the importance of plant hormones in abiotic stress, we analyzed the expression changes of DEGs involved in the plant hormone signal transduction. After 1-h salt treatment, DEGs related to signaling network of auxin: auxin influx carrier (AUX), auxin-responsive protein (IAA), gibberellin (gibberellin receptor, GID1), abscisic acid (protein phosphatase 2C, PP2C), and ABA responsive element binding factor (ABF) were highly up-regulated. While DEGs involved in ethylene (ethylene-responsive transcription factor 1, ERF1), brassinosteroid (BR-signaling kinase, BSK), and xyloglucosyl transferase (TCH) were down-regulated. On the other hand, the numbers of DEGs involved in plant hormone signal transduction were lower after 21-day salt treatment than those after 1-h salt treatment (Figure 6C). Soluble sugars play an important role in the osmotic adjustment of plant salt tolerance. Soluble sugar in leaves of *S. portulacastrum* increased 1.5 fold after 1 h of salt treatment but decreased 0.72 fold after 21 days of salt treatment (Figure 6E). These results were consistent with the expression trend of genes related to starch and sucrose metabolism in the transcriptome (Figure 6D).

**Figure 7:**
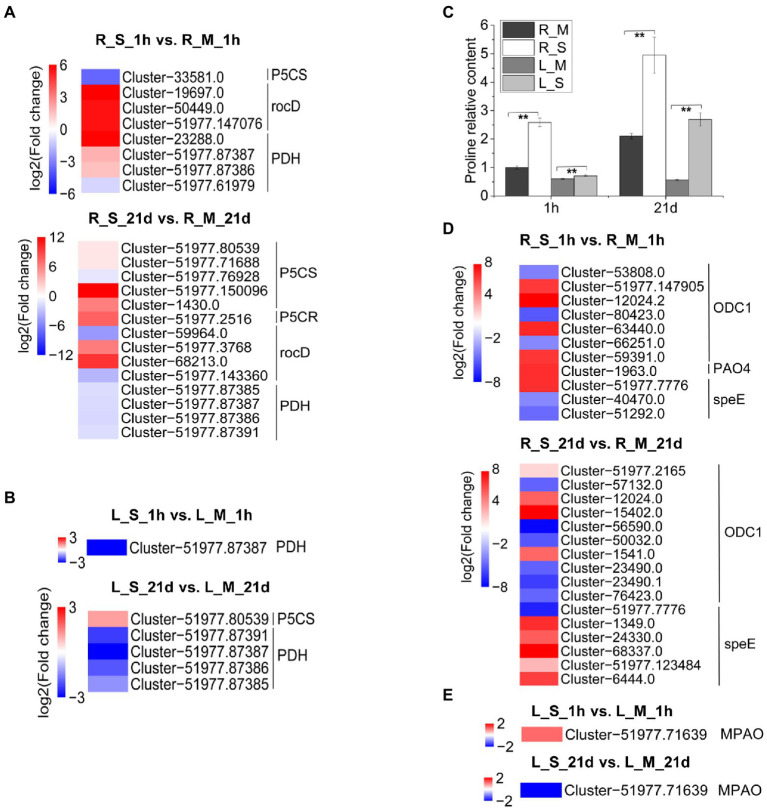
Expression analysis of DEGs involved in proline metabolism. **(A)** Expression analysis of DEGs in *S. portulacastrum* roots involved in proline metabolism after 1 h (R_S_1h vs. R_M_1h) and 21 days (R_S_21d vs. R_M_21d) of salt treatment (400 mM Na). **(B)** Expression analysis of DEGs in *S. portulacastrum* leaves involved in proline metabolism after 1 h (L_S_1h vs. L_M_1h) and 21 days (L_S_21d vs. L_M_21d) of salt treatment. **(C)** The relative content of proline in roots and leaves compared to the R_M sample. R_M and L_M mean roots and leaves under control condition. R_S and L_S mean roots and leaves under salt stress. The error bars were calculated from three biological replicates. Mean separation was based on Tukey HSD test at *p* < 0.01 (^**^) levels. **(D,E)** Expression analysis of DEGs in *S. portulacastrum* roots and leaves respectively involved in polyamines metabolism after 1 h and 21 days of salt treatment.

Our results showed that genes involved in proline metabolism were differentially expressed (Figures 7A,B) and proline content in *S. portulacastrum* significantly increased after 21-day salt stress (Figure 7C). Proline is an important osmolyte in higher plants, which is synthesized from glutamic acid and ornithine. ∆-1pyrroline-5-carboxylate synthetase (P5CS) and 1-pyrroline-5-carboxylate reductase (P5CR) catalyze the biosynthesis of proline from glutamic acid (Székely et al., 2008). In catabolism, proline dehydrogenase (PDH) degrades proline as pyrroline-5-carboxylate (P5C; Peng et al., 1996). In roots, the increased content of proline was mainly due to the up-regulated expression of *P5CS*, *P5CR* and ornithine-oxo-acid transaminase (*rocD*), which catalyzed the production of proline from ornithine. At the same time, the down-regulated expression of *PDH* might inhibit the degradation of proline (Figure 7A). The increase in proline content in leaves could be due to the inhibition of *PDH* expression (Figure 7B). Additionally, genes in regulation of polyamine metabolism were also differentially expressed (Figures 7D,E).

### Differential expression of transcription factors under salt stress

**Figure 8:**
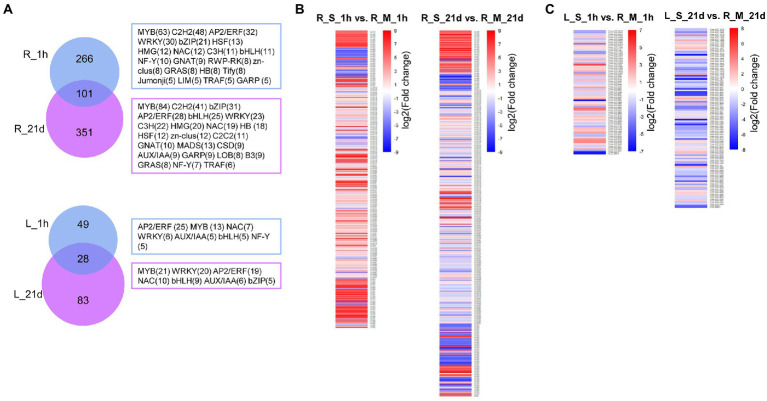
Analysis of differentially expressed TFs in roots and leaves of *S. portulacastrum.* **(A)** Venn diagrams for salt-induced TFs in roots and leaves of *S. portulacastrum.* R_1h and R_21d means differentially expressed TFs in roots after salt treatment (400 mM Na) for 1 h and 21 days. L_1h and L_21d means differentially expressed TFs in leaves after salt treatment after 1 h and 21 days. The TF families and gene numbers (*n* > 4) were shown in the right. **(B)** Expression analysis of the TFs in each comparison.

A large number of transcription factors (TFs) were differentially expressed after the exposure to Na. In roots of *S. portulacastrum*, 367 TFs and 452 TFs were differentially expressed 1-h and 21-day after salt treatment, respectively (Supplementary Table 6). The top five most differentially expressed transcription factors were genes in MYB, C2H2, AP2/ERF, WRKY, and bZIP families (Figure 8A). Compared with roots, the number of differentially expressed TFs in *S. portulacastrum* leaves was significantly lower than in roots. There were 367 and 452 DEGs in roots compared to 77 and 111 in leaves after 1 h and 21 days, respectively. The top five abundant TF families in leaves were AP2/ERF, MYB, WRKY, NAC, and bHLH (Figure 8A). The analysis of differentially expressed TFs showed that more TFs in the roots than leaves of *S. portulacastrum* after 1-h salt treatment, and their expressions were mostly up-regulated (Figure 8B). However, after 21 days of salt treatment, the number of down-regulated TFs increased significantly (Figure 8C).

### Identification of coexpression modules related to salt tolerance

**Figure 9:**
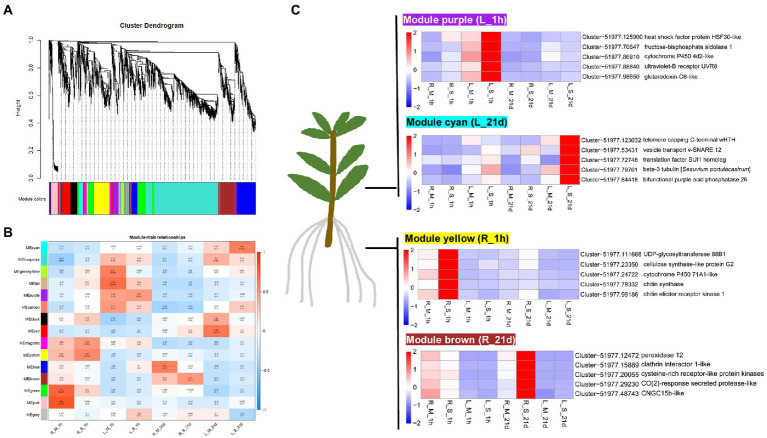
WGCNA analysis of genes in roots and leaves of *S. portulacastrum.* **(A)** Hierarchical cluster tree showing co-expression modules identified by WGCNA. Each leaf in the tree represents one gene. The major tree branches constitute 15 modules, labeled with different colors. **(B)** Module–sample association. Each row corresponds to a module, labeled with a color as in **(A)**. The numbers in each cell at the row-column intersection indicates the correlation coefficient between the module and the sample. **(C)** Heatmaps showing the selected hub genes in each module.

In order to gain a better understanding of the relationships among different genes implicated in *S. portulacastrum* responses to salt stress, the WGCNA analysis was carried out. The top 5000 DEGs with the average FPKM were divided into 15 modules, which were presented in the cluster dendrogram (Figure 9A). Each module was marked with a different color. Further correlation analysis between the modules and the samples was carried out (Figure 9B). The results showed that the yellow module, with 431 identified genes, was highly associated with the R_S_1h sample (Supplementary Table 7). The brown module (438 genes) was highly associated with R_S_21d sample. The purple module, representing 144 genes, was highly correlated with L_S_1h sample. The cyan module, containing 45 genes, was highly related to the L_S_21d sample.

The WGCNA was also be used to screen the hub genes in the module based on the KME (eigengene connectivity) value. The hub gene means a high degree of connection with the genes in the module, which may play an important role in the salt tolerance of *S. portulacastrum.* Heatmap showed that the yellow module-specific genes were over-expressed in R_S_1h (Figure 9C). Cytochrome P450 71A1, UDP-glycosyltransferase 88B1, cellulose synthase-like protein G2, chitin elicitor receptor kinase 1, and chitin synthase were identified as candidate hub genes in this module. The brown module genes were over-expressed in R_S_21d (Figure 9C). Genes encoding peroxidase 12, cysteine-rich receptor-like protein kinase, clathrin interactor 1, CO (2)-response secreted protease, and CNGC15b were identified as candidate hub genes for this module. The purple module genes were over-expressed in L_S_1h (Figure 9C). Glutaredoxin-C6, fructose-bisphosphate aldolase 1, ultraviolet-B receptor UVR8, heat shock factor protein HSF30, and cytochrome P450 4d2 were identified as candidate hub genes in this module. The cyan module genes were over-expressed in L_S_21d (Figure 9C). Genes encoding bifunctional purple acid phosphatase 26, vesicle transport v-SNARE 12, telomere capping C-terminal wHTH, translation factor SUI1 homolog, and beta-3 tubulin were identified as candidate hub genes for this module.

## Discussion

Land salinization is one of the most important environmental problems in the world. It was estimated that about 10% of the land surface and 50% of irrigated land have been affected by salinization (Ruan et al., 2010). Annual losses due to salt-affected land in agricultural production are more than US$12 billion and rising (Flowers et al., 2010). Salinity is now a key restraint to crop productivity (Shahzad et al., 2019). On the other hand, world food production is projected to increase between 50% and 70% by 2050 to match the estimated growth of population (Shabala et al., 2013). It is certain that the current arable land is far less sufficient for crop production. A solution to expand crop production is to use halophytic plants to remediate soil salinity and convert the soils to cultivable land (Karakas et al., 2020; Munir et al., 2022). Thus, an in-depth understanding of halophytic plant tolerance to salt stress would enable us to better use this group of plants for remediation of saline soils. *Sesuvium portulacastrum* is a typical halophyte, but its salt tolerance mechanisms remain largely unclear. Our study showed that *S. portulacastrum* plants when exposed to 400 mM Na were able to produce shoot fresh weight and root numbers comparable to those of control plants (Figure 1). The ability of *S. portulacastrum* to thrive in such a higher concentration of Na was elucidated through RNA-Seq and qRT-PCR analyses. Our results showed that *S. portulacastrum* has developed multiple strategies to cope with saline growth conditions.

### Regulation of cellular Na^+^/K^+^ homeostasis

Maintaining a proper intracellular K^+^/Na^+^ ratio is critical for plants to tolerate salt stress. *Sesuvium portulacastrum* is able to deliberately control K^+^ and Na^+^ levels when grown under high salt stress. Compared with the control samples, higher content of Na^+^ was detected in the leaves and roots of *S. portulacastrum* after 21 days of salt treatment. At the same time, the K^+^ content in the samples of the treatment group was significantly reduced (Figures 4A,B). Like other halophytes, *S. portulacastrum* can maintain a higher Na^+^/K^+^ ratio in roots and leaves. This is likely due to the expression of ion transport related genes. We have identified a large number of ion transport related genes that were differentially expressed in *S. portulacastrum* under salt stress. The expression of *CNGC* genes in roots after 1 h of Na treatment was up-regulated (Table 1). This suggests that *CNGC* gene was involved in Na uptake in the early stage of salt stress. After 21 days of salt treatment, the expression of *CNGC* was down-regulated, which could inhibit Na uptake at the later stage of salt stress (Figure 4C). Recent studies have shown that increased transcript levels of a few *AtCNGCs* in *Arabidopsis* roots or shoots after exposed to high levels of NaCl (Duszyn et al., 2019). For example, *AtCNGC20* was up-regulated in roots after exposure to Na; *AtCNGC5* and *AtCNGC17* were reported to be implicated in salt tolerance (Guo et al., 2008; Mian et al., 2011; Ladwig et al., 2015; Massange-Sánchez et al., 2016; Vadim, 2018). Our speculation is that ion transporter, such as CNGCs regulate Na absorption, while NHXs may play an important role in control of Na accumulation. From our result, NHX and H^+^-ATPase genes were up-regulated in *S. portulacastrum* root under salt stress (1 h and 21 days). NHX is one of the important families participating in Na^+^ extrusion and sequestration. NHXs located on the plasma membrane of the cell, such as NHX7/SOS1 can exclude Na^+^ from the root system (Ji et al., 2013). In addition, NHX1 and NHX2 located on the vacuole membrane may participate in the compartmentalization of Na^+^ in the vacuole and the K^+^ balance in the vacuole (Jiang et al., 2010). At the same time, proton pump (H^+^-ATPase) located on the plasma membrane of the cell provided power for Na^+^ extrusion and sequestration (Gaxiola et al., 2007). In addition, several ion transport related genes in *S. portulacastrum* had been reported. Previous studies found that heterologous overexpression of *SpSOS1* and *SpAHA1* genes improved salt tolerance in yeast and *Arabidopsis* (Zhou et al., 2015, 2018). These genes were also identified in our study (Supplementary Table 5). In addition, more ion transport related genes were identified in our study, such as *AKT* and *HKT*. The expression of *AKT* and *HKT* in roots of *S. portulacastrum* was down-regulated after salt stress. But in halophytic turf grass, *SvHKT1;1* gene was up-regulated in response to high concentrations of NaCl (500 mM), (Kawakami et al., 2020). This is in contrast to the results in the *S. portulacastrum*. We think that *CNGCs* and *NHXs* are important gene families implicated in the regulation of Na^+^ uptake, extrusion, and sequestration, and their coordinate actions could maintain Na^+^/K^+^ at appropriate ratios and lead to Na tolerance in *S. portulacastrum*. Further research is warranted to confirm these propositions.

### Changes in osmolytes

The loss of water due to the decrease in osmotic pressure is one of the great constraints faced by plants growing in saline soils. Accumulation of compatible osmolytes is an important strategy for plants to cope with osmotic stress (Apse and Blumwald, 2002). Different species have different osmolyte profiles under salt stress. Halophytes usually accumulate one or more than one compatible osmolytes, such as proline, glycine betaine, and sorbitol. Previous studies showed that under salt and drought conditions, the proline content in the callus and axillary buds of *S. portulacastrum* significantly increased (Lokhande et al., 2010). Our study also found that, compared with the control samples, the proline content in *S. portulacastrum* roots and leaves significantly increased under short-term (1-h) and long-term (21-day) salt treatments (Figure 7C). According to transcriptome data, we found that the accumulation of proline in roots depends on the up-regulated expression of genes related to proline synthesis (P5CS, P5CR, rocD) on the one hand, and on the other hand from the down-regulated expression of genes related to degradation (PDH; Figure 7A). However, the accumulation of proline in leaves is more dependent on the inhibition of degradation-related genes (Figure 7B). Soluble sugar is another important osmolyte under salt stress, which can protect specific macromolecules or sustain membrane stability (Bartels and Sunkar, 2005). Other studies have demonstrated that halophyte *Thellungiella* under salt treatment accumulated high content of soluble sugars (Wang et al., 2013). From the result of KEGG analysis, the pathway of starch and sucrose metabolism was enriched in leaves of *S. portulacastrum* under salt stress (Figures 6A,B). Further study showed that the soluble sugar content in leaves of *S. portulacastrum* increased after 1 h of salt stress but decreased after 21d of salt stress (Figure 6E). Thus, proline and soluble sugar may play an important role in maintaining the osmotic balance of *S. portulacastrum* in a high-salt environment.

Polyamines play significant roles in regulating plant defense responses to various environmental stresses, including salt stress, metal toxicity, and oxidative stress (Zhong et al., 2020). Exogenous application of putrescine (Put) improved plant salt tolerance (Chattopadhayay et al., 2002; Gill and Tuteja, 2010). In plants, the first rate-limiting reaction of Put synthesis is either arginine catalyzed by arginine decarboxylase (ADC) or ornithine catalyzed by ornithine decarboxylase (ODC). In this study, after 1 h of salt stress, the expression of several *ODC* genes in the root of *S. portulacastrum* increased, which catalyzed the biosynthesis of putrescine from ornithine. The expression of polyamine oxidase 4 (*PAO4*) was up-regulated (Figure 7D). After 1 h of salt stress, the polyamine oxidase gene *MAPO* was up-regulated in leaves of *S. portulacastrum* (Figure 7E). *PAO4* is able to catalyze the biosynthesis of putrescine from spermine and spermidine (Alcázar et al., 2011). After 21 days of salt stress, the expression of spermidine synthase genes (*speEs*) in *S. portulacastrum* was up-regulated, which catalyzes putrescine to spermine and spermidine (Figure 7D). This suggests that the balance of polyamines plays a role in the salt tolerance of the *S. portulacastrum.*

### ROS homeostasis

Salt stress can induce excessive ROS accumulation in plants which leads to cell damage and cell death. The tolerance of halophytes to high salinity depends to a certain extent on their ability to maintain ROS homeostasis (Jayakumar et al., 2014). Synthesis of antioxidant metabolites is an important strategy for plants to eliminate excess ROS under high salt stress. Ascorbate and GSH are important antioxidant metabolites in plants. Halophytes are found to have a more efficient ascorbate-glutathione cycle. For example, the ascorbate and GSH content of halophyte *Lycopersicon pennellii* was significantly higher than that of glycophyte relative *L. esculentum* (Shalata et al., 2001). However, the content of GSH in *S. portulacastrum* decreased after 1-h of salt treatment (Figure 5D). This may be due to the oxidation of GSH to GSSG. RNA-Seq data also showed that the expression of genes involved in catalyzing the oxidation of GSH into GSSG was significantly up-regulated (Figure 5C). Another important way to scavenge ROS in halophytes is through enzymatic agents, such as SOD (Grene et al., 2002), CAT (Willekens et al., 2014), APX (Shigeru et al., 2002), and POX (Fagerstedt et al., 2010). From the WGCNA analysis of this study, POX was identified as one of the hub genes in the salt-stressed modules in roots (Figure 9C). POXs are isoenzyme that removes H_2_O_2_ in the outer plastid space (Fagerstedt et al., 2010). Previous studies showed that increased activity of POXs was found in halophytes, such as *Cakile maritima* and *Hordeum marinum* (Seckin et al., 2010; Ellouzi et al., 2011). Our results also indicated that the expression of POX genes was up-regulated in roots of *S. portulacastrum* under salt stress (Figure 9C). Therefore, we believe that the GSH/GSSH cycle and POX play an important role in the scavenging ROS induced by salt in *S. portulacastrum*.

### Changes in tissue-special gene modules

Another finding in this study is tissue-special gene modules identified by WGCNA analysis (Figure 9). WGCNA analysis aimed to identify co-expressed gene modules and to explore the core genes in the modules, through which a hierarchical clustering tree was constructed by calculating the correlation coefficient between any two genes. Different branches of the clustering tree represent different gene modules, and different colors represent different module. Based on the weighted correlation coefficients of genes, genes were classified according to their expression patterns, and genes with similar patterns were grouped into one module. The identification of hub genes in relation to modules provided a new and important perspective for understanding the salt tolerance mechanism in *S. portulacastrum*. In roots of *S. portulacastrum*, the expression of genes related to lignin synthesis in the phenylpropane metabolic pathway (cytochrome P450 71A1-like), cellulose synthesis (cellulose synthase-like protein G2), endocytosis (clathrin interactor 1-like), and perceive CO_2_ concentration to regulate the pathway of stomatal development CO_2_-response secreted protease-like) were up-regulated. An important adaptation of plants to salt stress is differential regulation of growth, accompanied by dynamic changes and rearrangement of plant cell walls, and cellulose and lignin were the structural composition of cell walls (Cosgrove, 2015; Tenhaken, 2015; Yan et al., 2021). This may indicate that the strong cell wall regulation ability of *S. portulacastrum* enhances its salt tolerance. In leaves of *S. portulacastrum*, the expression level of vesicle transport related genes (vesicle transport v-SNARE 12) and tubulin (beta-3 tubulin) were up-regulated under salt stress. Vesicle transport and tubulin play important roles in plant salt tolerance. Under high salinity conditions, regulating the reorganization of cortical microtubules is the key to the survival of plant cells (Endler et al., 2015). In *Arabidopsis*, ethylene signal promotes the reorganization of cortical microtubules induced by salt stress (Dou et al., 2018). This inspired us to think what role does tubulin play in promoting salt tolerance in halophytes? These questions will be part of our follow-up research.

The mechanisms of salt tolerance in halophytes have been continuously studied over the years. Most studies focus on osmotic protection and ion homeostasis. Some halophytes maintain Na^+^/K^+^ balance through salt secretion. For example, in halophyte quinoa, salts are deposited in epidermal bladder cells (EBCs; Kiani-Pouya et al., 2017). In *S. portulacastrum*, the maintenance of Na^+^/K^+^ balance is mainly dependent on the storage of the excess Na in the vacuole. Halophytes maintain a better Na/K ratio by removing Na from the cytoplasm, transporting Na from root cells to the xylem, and sequestrating Na in the vacuole in which *SOS1*, *HKT1*, and *NHXs* ion transporters play important roles (Flowers et al., 2015). In this study, more ion transport related genes (*GLRs*, *CNGCs*, *AKTs*, *HAKs*, *KEAs*, *KUPs* and *TPKs*) were identified in response to salt stress. In addition, the results of WGCNA analysis suggest that cell wall formation, vesicle transport and tubulin may play significant roles in salt tolerance in *S. portulacastrum*.

## Conclusion

**Figure 10:**
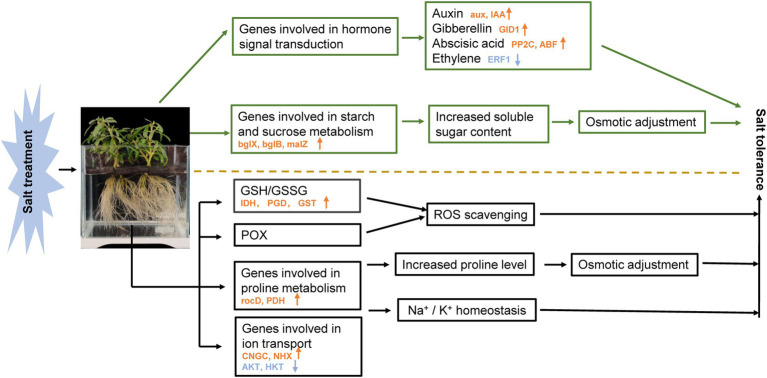
Schematic presentation of the potential mechanism of salt stress tolerance in *S. portulacastrum.* The yellow arrows in the figure represent the increased expression of related genes. Blue arrows represent decreased gene expression. The mentioned genes were selected from plants after 1 h of Na treatment.

*Sesuvium portulacastrum* is a halophytic species and has been considered a valuable plant to be used for phytoremediation of saline soils. After 400 mM Na treatment, Na contents in roots and leaves were much higher than K contents in *S. portulacastrum*. A large number of DEGs were detected from RNA-Seq data. A variety of ion transport related genes (*CNGCs, NHXs, AKTs, HKTs*) are implicated in Na uptake and accumulation, of which *CNGCs* could be involved in Na uptake, and *NHXs* could be responsible for controlling Na extrusion and sequestration. Their coordinated action could maintain Na^+^/ K^+^ homeostasis in the *S. portulacastrum*. Soluble sugar and proline play an important role in the osmotic regulation of *S. portulacastrum* under salt stress. Glutathione metabolism and POX participated in scavenging reactive oxygen species under salt stress. Additionally, transcription factors and plant hormones including auxin, gibberellin, abscisic acid, and ethylene are involved in regulating the salt tolerance of *S. portulacastrum*. Our study shows that *S. portulacastrum* has developed a suite of mechanisms for accumulation and tolerance of Na, suggesting that it could be an important species to be used for remediation of saline soils (Figure 10).

## Data availability statement

The datasets presented in this study were deposited in the NCBI repository with an accession number of PRJNA848266, available at: https://www.ncbi.nlm.nih.gov/bioproject/PRJNA848266.

## Author contributions

DW: conceptualization, data curation, formal analysis, investigation, methodology, and writing–original draft. NY: data curation, methodology, software, and visualization. CZ: methodology. WH: methodology. GY: methodology and software. JC: conceptualization and writing–review and editing. XW: conceptualization, funding acquisition, project administration, resources, supervision, and writing–review and editing. All authors contributed to the article and approved the submitted version.

## Funding

The authors are thankful to the National Natural Science Foundation of China (32102331), the Natural Science Foundation of Fujian Province (2022J011141 and 2020J01867) and Department of Education, Fujian Province (JAT200426) for providing the financial support.

## Conflict of interest

The authors declare that the research was conducted in the absence of any commercial or financial relationships that could be construed as a potential conflict of interest.

## Publisher’s note

All claims expressed in this article are solely those of the authors and do not necessarily represent those of their affiliated organizations, or those of the publisher, the editors and the reviewers. Any product that may be evaluated in this article, or claim that may be made by its manufacturer, is not guaranteed or endorsed by the publisher.
